# An Abridgement of Mr. Marson's Papers on Vaccination

**Published:** 1807-07

**Authors:** 


### *An* Abridgement *of Mr.* Marson's Papers *on* Vaccination.

THE address of yours to Correspondents in No. 98, relative to the partial suspension of Communications on the subject of Vaccination, I did not think would attach to me; but on the receipt of your last Number, on finding my letter to you omitted, I think it necessary to ask your attention to a few words.

In the month of October last, (you say November) I wrote a second paper on vaccination; this paper you did not insert till February; the succeeding month Mr. Ring was pleased to honour me with a direct notice, and at the same time complimented me on my "very candid style," though he "unfortunately differed from me." The arguments of Mr. Ring I could not think of that solidity as to refute mine, and occasioned the letter I speak of. Mr. Ring has ever been fortunate in the immediate publication of his letters, however prolix. I am one of those long acquainted quainted with variolous inoculation, with which I have compared the vaccine. I observed when vaccination was first broached, it fell into hands that never inoculated before; they soon imagined, in the mildness of the cases, that a puncture would do the business, and never conceived any thing further was wanting. I do assure you, gentlemen, that much is wanting to make vaccination complete; and it is from this imperfection of our knowledge that the disputes and cavils have arisen between the theorist and experienced practitioner.

It is the opinion of Dr. Willan, in his publication, that "the employment of recent lymph seems no essential requisite, for the disease is as often produced by dried as by fluid matter, though the former may be more liable to fail." Admitting then that practitioners used only dry matter, I think, after the above opinion, Dr. Willan would never be brought to say it entirely depended on the aridity of the matter that these inoculations so frequently failed; and as to the taking it at an early period, I think I shall make it appear from Mr. Ring's own observations, that the neglect in not so doing, shall not better account for the failure of inoculation than the two preceding circumstances.

Mr. Ring, in controverting Dr. Clarke's idea, (Medical Journal, vol. xvi. p. 319) respecting the difference of a vesicle and a pustule, says, he is inclined to think that it is merely an inspissation of the fluid. This inspissation I am of opinion is a proof of the vaccine virus being more concentrated, and accordingly the two practitioners, on the ground of genuine matter, had the advantage of Mr. Ring, provided they used only matter taken in this concentrated state.

I have nothing to do with decomposed or diluted matter, my argument was built on the proviso the matter was *good*, that no particular address was required in performing the operation, concurring with Mr. Ring, that if the smallest quantity of this good matter was inserted or absorbed, the inoculation would take place.*

I do not mean to divert the reader's attention from the subject by observations on the words "convenient" and "comfortable;" but in this, as well as in other remarks of Mr.

* On this point Mr. M. observes, in a note, that Mr. Ring never imputes failure to insusceptibility in the patient. As Mr. R. writes for *practitioners*, and not for *theorists*, it was unnecessary to allude to that cause of failure.

Mr. Ring, we perceive a hurried judgment, for in my advising regimen and medicine,* I take upon myself a greater responsibility than if I confined myelf to the practice of Dr. Jenner.

Mr. Ring says, my observations on the breaking up of a vesicle, and irritation of the arm, are rather obscure and unintelligible; they may be so, but I have the pleasure of observing that I perfectly comprehend him; as, for example, he never says a word about the patient "being before healthy," in the case I alluded to, see vol. xvi. p. 318; but now it seems *convenient* to bear him out in his argument; yet I am apprehensive this healthy patient, instead of being a comfort to him, will be as a twig to a drowning man, for who does not know that in vigorous health there is the greater inflammatory tendency; the vesicle there being broken, and the arm irritated as described, we have something in vigorous health to apprehend, particularly when generous living is allowed and there is a costive habit; but in reduced health, where regimen and medicine are prescribed previous to or at the time of the inoculation, there is not much, I think, to be afraid of; indeed, Mr. Ring says, "the shield of Jenner generally protects them," even in the most vigorous health, vol. xvi. p. 318. I believe, when inflammation had taken place from a broken vesicle and irritation of the arm in this vigorous healthy patient, Mr. Ring would call in regimen and medicine to his assistance, and lament he had not, by these means, previously prepared the patient for the fatal mischief he describes; and I believe Dr. Willan would support me in this opinion.

If Mr. Ring disagrees with me respecting a good habit, I am happy to say he accords with me as to a bad one; he says, most people will agree with Dr. Willan's implication, that it would be well to attend to the constitution of the patient previous to or at the time of inoculation; this attention is what I argue for, medicine and regimen, though medicine is denounced as unnecessary in vaccination, in one of the Reports of the Jennerian, Society, of which Mr. Ring has the honour to be a Vice President. However, we find now, that regimen and medicine have Mr. Ring's sanction in bad habits; and it leads us to conclude that they cannot be amiss in vigorous healthy ones, where there

* The public are sick of regimen and medicine, when there is no occasion for them.

there is great inflammatory tendency, as in Mr. Smart's case: for example, though it is not exactly clear to me that it was owing to vaccination, I have met with an erysipelas peculiar to young children at the breast, which very much corresponds with the symptoms he describes; but you, gentlemen, in your Note, vol. xvii. p. 158, say, that you have "no doubt the inoculation was the exciting cause of all the mischief," though at the same time you wish Mr. Smart "had been more minute in his account of the cause, manner, and degree of injury inflicted."

There is something very remarkable in the pulsation, that it should commence *so soon* after the inoculation, as witnessed by the child's mother; that it should be visible in every inflammation; that it is not always a transient visitor, but is to be met with when the pustule is broken; that this pulsation should prove a constitutional affection, and yet that this very conspicuous constitutional pulsation should never be introduced to Dr. Jenner or Dr. Willan, for I do not find it characterized by either of them. The above is the pulsation of Mr. Ring, that I mean is not observable at a very early period of the inoculation, though when I perceive it I assure myself of constitutional affection; I say, when I perceive the pulsation, because it would appear there is sometimes a well formed pustule and circumscribed areola without a pulsation. This may startle Mr. Ring; but will he say there can be a local cow-pock, as reported by the Jennerian Society, if that cow-pock has the pulsation he says every inflammation has, and that this pulsation is a proof of the local cow-pock being a constitutional affection?

I hope what I have said will remove the prejudices of Mr. Ring, and that he will give what I propose a practical attention; my wish is, as well as his, to serve the cause of vaccination; and as he is persuaded it is not yet brought to perfection, he should not be too hasty in condemning what he never made a trial of. I would recommend him to indiscriminately set apart twenty patients; ten I would inoculate in the way I propose, with diet and regimen; the other ten according to his own plan; and then candidly tell us the general result.

*Worksop, Nottingham, March* 12, 1807.

